# Factors affecting patient outcome in primary cutaneous aspergillosis

**DOI:** 10.1097/MD.0000000000003747

**Published:** 2016-07-01

**Authors:** Alexander M. Tatara, Antonios G. Mikos, Dimitrios P. Kontoyiannis

**Affiliations:** a Department of Bioengineering, Rice University; b Department of Infectious Diseases, The University of Texas MD Anderson Cancer Center, Houston, TX.

**Keywords:** aspergillosis, cutaneous, fungal infection, infectious diseases

## Abstract

Primary cutaneous aspergillosis (PCA) is an uncommon infection of the skin. There is a paucity of organized literature regarding this entity in regard to patient characteristics, associated *Aspergillus* species, and treatment modalities on outcome (disease recurrence, disease dissemination, and mortality).

We reviewed all published reports of PCA from 1967 to 2015. Cases were deemed eligible if they included the following: patient baseline characteristics (age, sex, underlying condition), evidence of proven or probable PCA, primary treatment strategy, and outcome.

We identified 130 eligible cases reported from 1967 to 2015. The patients were predominantly male (63.8%) with a mean age of 30.4 ± 22.1 years. Rates of PCA recurrence, dissemination, and mortality were 10.8%, 18.5%, and 31.5%, respectively. In half of the cases, there was an association with a foreign body. Seven different *Aspergillus* species were reported to cause PCA. Systemic antifungal therapy without surgery was the most common form of therapy (60% of cases). Disease dissemination was more common in patients with underlying systemic conditions and occurred on average 41.4 days after PCA diagnosis (range of 3–120 days). In a multivariate linear regression model of mortality including only patients with immunosuppressive conditions, dissemination and human immunodeficiency virus/acquired immune deficiency syndrome were statistically significantly associated with increased mortality.

Nearly one-third of patients with PCA die with the disease. Dissemination and host status are critical in patient outcome.

## Abstract 2

Supplemental Digital Content is available in the text

## 1 Introduction

Primary cutaneous aspergillosis (PCA) is a rare but life-threatening invasive fungal infection of the skin caused by the mold *Aspergillus*. It is defined as aspergillosis in which an infected skin lesion is the initial source of disease.^[1]^ This is in contrast to secondary cutaneous aspergillosis, the more common form, which involves the hematogenous spread of *Aspergillus* infection from a distal site (e.g., the lungs) to the skin. Particularly in immunocompromised patients, PCA has the potential to progress to systemic infection (disease dissemination) and is frequently lethal.^[1,2]^ As the population of immunocompromised patients is expanding, it is expected that PCA will also rise in prevalence.^[2]^ Due to the challenges associated with diagnosing fungal disease, especially in complex patients with multiple comorbidities, it is likely that PCA is underdiagnosed and underreported in the literature.^[3,4]^

Due to the rarity of the disease, the patient characteristics, rates of disease recurrence, dissemination, and mortality are understudied. To that end, we undertook a systematic review of the epidemiological, clinical, diagnostic, and therapeutic aspects of this serious infection with particular emphasis on the underlying condition, associated species of *Aspergillus*, primary treatment strategy, and rates of recurrence, dissemination, and mortality. Our underlying hypothesis is that factors such as host condition and disease dissemination may significantly affect patient outcome in PCA.

## 2 Methods

A MEDLINE search using the phrase “primary cutaneous aspergillosis” was conducted as recently as March 12, 2016. All English-language manuscripts from this search were then reviewed for cases of PCA. Only cases that provided the following information were included in this analysis: patient baseline characteristics (age, sex, underlying condition), evidence of proven or probable PCA^[5]^ with no indications of primary infection at another site, primary treatment strategy, and patient outcome (mortality with disease). As a systemic review and analysis of the literature, no institutional review board approval was required for this study.

### 2.1 Database development

JMP Pro 11.0.0 (SAS Institute Inc, Cary, NC) was utilized to build a database of patients eligible as described earlier. The following information was recorded in the database as was available in the literature: year of publication; patient age at presentation, underlying condition (further divided into local underlying condition, systemic underlying condition, and no underlying condition), and patient sex; diagnosis modality; associated *Aspergillus* species; noted association with a foreign body (e.g., catheter, wound dressing); primary treatment method (none, antifungal, surgical, antifungal, or combination); occurrence of disease recurrence, dissemination, and mortality with disease; time from appearance of disease until mortality; dissemination diagnosis modality, time from PCA diagnosis to dissemination diagnosis, and site of dissemination. We excluded cases in which the age, sex, or underlying host condition was unclear; cases in which diagnosis could not be confirmed; case reports with diagnoses but no discussion of treatment and outcome; and patients lost to follow-up before outcome was noted.

### 2.2 Statistical analyses

JMP Pro 11.0.0 was utilized for all statistical analyses performed. For univariate analysis, categorical data were compared by a 2-tailed Fisher's exact test (α = 95%). To study which factors affect mortality in immunocompromised patients, a multivariate linear regression model was built with the inclusion of all variables that had *P* < 0.2 in univariate analysis.^[6]^ Patients with no known immunodeficiencies in the database were excluded from this model.

## 3 Results

Out of 113 manuscripts resulting from the literature search, 78 contained eligible patients, resulting in a total of 130 unique eligible PCA cases.^[7–84]^ The cases were reported from the years 1967 to 2015 (Supplementary Table 1). Most of the manuscripts consisted of single case reports or small case series. When manuscripts reported multiple patients, most often these patients shared the same risk factor (blood malignancy, stem cell transplantation [SCT], etc.) and occasionally the same species (particularly in cases of outbreak).^[9]^

### 3.1 Patient characteristics

The majority of the patient population was male (63.8%) (Table 1). The mean, median, and standard deviation were 30.4, 30, and 22.1 years of age, respectively. Ages ranged from neonatal to 81 years. The majority (61.5%) of patients were adults (between 18 and 65 years of age).

**Table 1:**
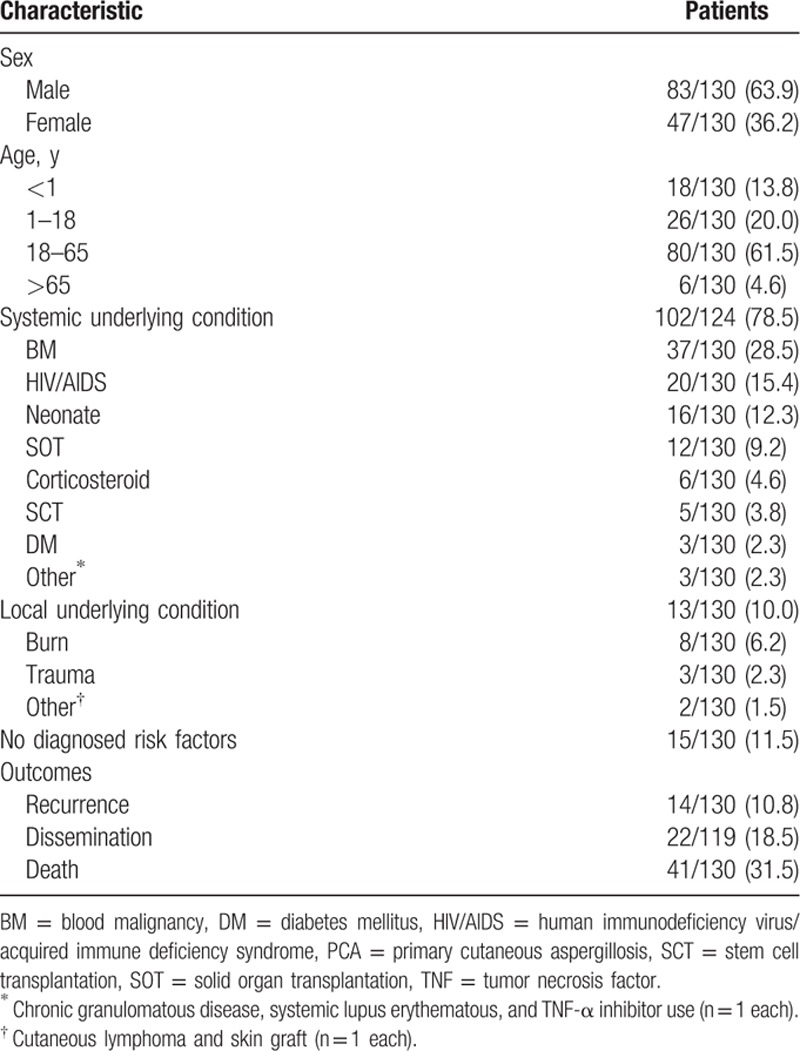
Characteristics of patients reported with PCA.

A variety of underlying conditions were encountered, the most common being blood malignancy (28.5%), followed by human immunodeficiency virus (HIV)/acquired immune deficiency syndrome (AIDS) (15.4%) and neonatal status (12.3%). A total of 11.5% of cases were reported with no diagnosed risk factors (presumably immunocompetent). Other risk factors included solid organ transplantation (9.2%), burns (6.2%), corticosteroid use (4.6%), SCT (3.8%), and diabetes mellitus and trauma (2.3% each). In the 3 patients with diabetes, poor control was suggested.^[44,58]^ In 64 of 130 cases (49.2%), the infection was noted to be associated with a foreign body (most commonly catheters or wound dressings). In the 10 adults (ages 18–65) with no diagnosed immunodeficiencies or risk factors, occupational information was provided in 5 of 10 cases. Four of these patients were agricultural workers^[18,42,73,76]^ and 1 was a researcher in a laboratory studying *Aspergillus* infection.^[79]^ Infection was noted to be preceded by minor trauma in 3 of 10 of these cases.^[73,76,79]^

Outcomes appear to be associated with specific patient characteristics (Table 2). Compared to patients from all other age groups, adults were significantly more likely to have disease recurrence (16%, *P* = 0.0095). Patients with an underlying condition were more likely to suffer from disease dissemination (24.7%, *P* = 0.0035) and death (39.2%, *P* = 0.0056). Patients with diabetes mellitus were significantly associated with increased disease recurrence (66.7%, *P* = 0.0335), blood malignancy was significantly associated with disease dissemination (34.4%, *P* = 0.0395), and SCT was significantly associated with both disease dissemination (80.0%, *P* = 0.0066) and death (80.0%, *P* = 0.0407). In 24 of 41 (58.5%) cases of fatal PCA, information pertaining to time from PCA appearance to death was included. The median, mean, and standard deviation were 46.4, 79.1, and 99.27 days, with a range of 3 to 420 days.

**Table 2:**
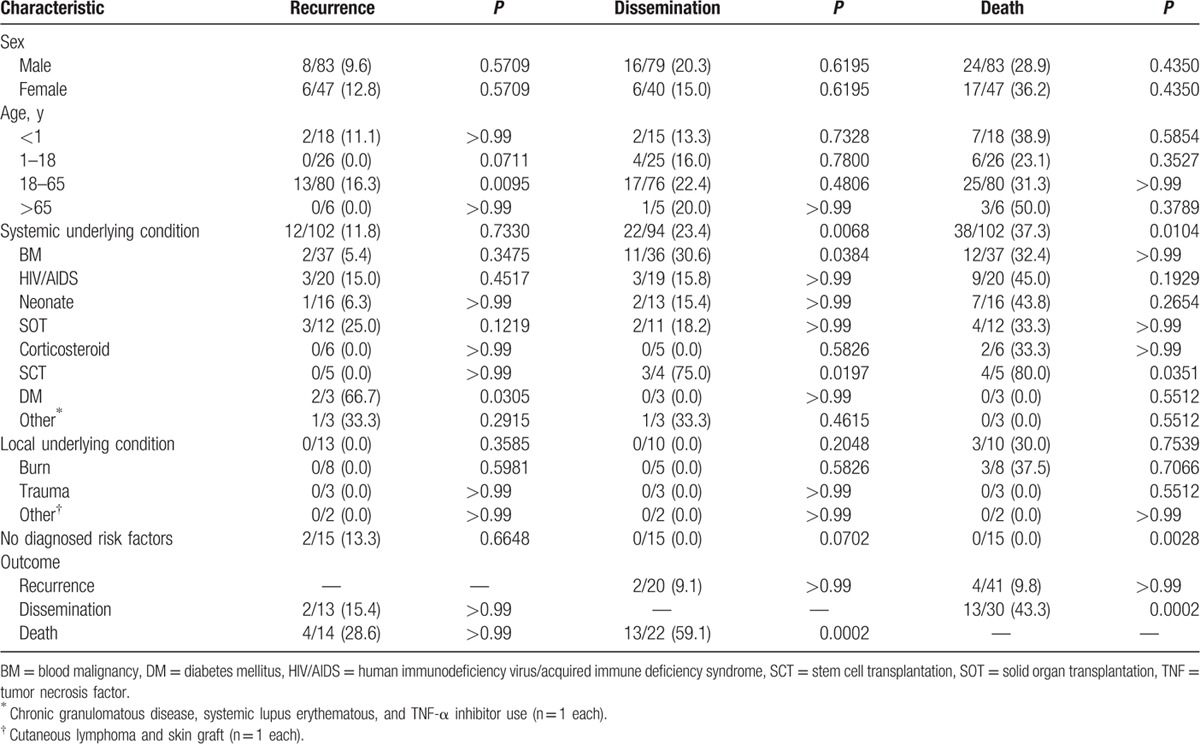
Patient characteristics and relationships with outcomes (univariate analysis).

### 3.2 *Aspergillus* species

In 111 of 130 (85.4%) reported cases, the associated species of *Aspergillus* was identified with a total of 7 unique species (Table 3). In 1 case, a coinfection with both *A flavus* and *A terreus* was reported.^[80]^ Out of the cases in which the species was identified, the most commonly reported species were *A fumigatus* (42.3%), *A flavus* (35.1%), and *A niger* (10.8%). In addition to the species reported in Table 3, *A glaucus* was identified in 1 case but was excluded from this analysis as the patient was lost to follow-up.^[85]^ Compared to other species, *A ustus* was significantly associated with greater mortality (80%, *P* = 0.0341). Particularly in immunocompromised patients, it was not unusual to have concomitant infections such as concurrent dissemination (*Mycobacterium tuberculosis*,^[8]^*Escherichia coli* and *Pneumocystis jirovecii*,^[36]^ cytomegalovirus,^[40]^ and *Mycobacterium avium* complex),^[56]^ localized PCA during dissemination of other pathogens (*M avium* complex,^[40]^*Pseudomonas aeruginosa*, and cytomegalovirus),^[77]^ and localized coinfection (*Staphylococcus capitis*).^[12]^

**Table 3:**
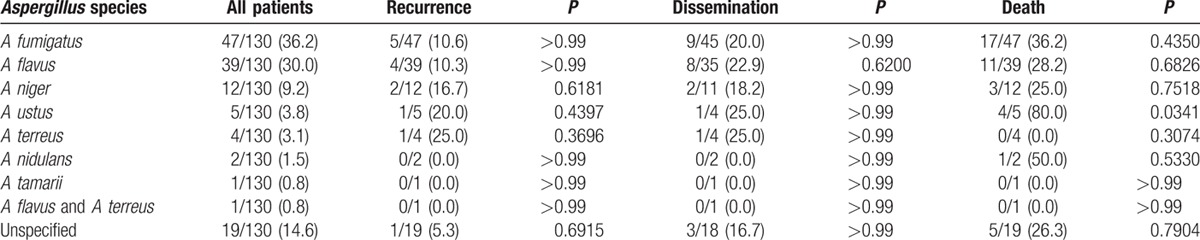
Associated *Aspergillus* species and relationships with outcomes (univariate analysis).

### 3.3 Primary treatment strategy

The most common treatment strategy was systemic *Aspergillus*-active antifungal therapy with no surgery (60.5%), followed by antifungals in combination with surgery (21.8%), surgery alone (12.9%), and no treatment (4.8%) (Table 4). Compared to the other treatment modalities, surgery alone was significantly associated with increased disease recurrence (37.5%, *P* = 0.0022) and a combination of surgery and systemic *Aspergillus*-active antifungal therapy was significantly associated with decreased mortality (13.8%, *P* = 0.0230). Patients receiving no treatment had greater mortality (71.4%, *P* = 0.0318).

**Table 4:**
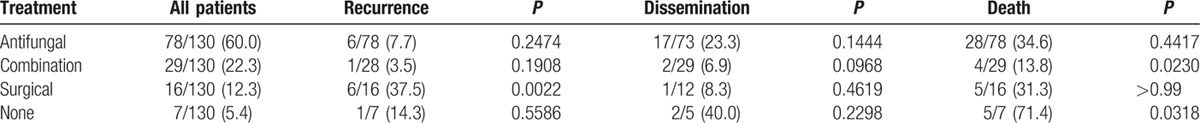
Primary treatment strategy and relationships with outcomes (univariate analysis).

### 3.4 Dissemination

In instances in which dissemination was described, the modality for making a diagnosis of PCA dissemination was reported in 20 of 22 (90.9%) cases and included histological analysis through means such as biopsy or sputum culture (50.0%), chest x-ray (25.0%), computed tomography (10.0%), and a high index of clinical suspicion (10.0%).^[16,39]^ The site of dissemination was described in 20 of 22 cases. Of these, the most common site of dissemination was the lungs (70.0%). In two cases, dissemination was reported at multiple organ sites: lungs, pericardium, stomach, liver, thyroid, gland, and brain in one patient;^[41]^ and lung, spleen, and mesentery in the other.^[62]^ In individual cases, there were reports of dissemination to the bone marrow^[8]^ and kidney.^[50]^ The time to dissemination (time from initial PCA diagnosis to diagnosis of dissemination) was reported in 10 of 22 cases (45.5%) and varied widely with a range of 3 to 120 days. The mean, median, and standard deviation were 41.4, 26.5, and 39.8 days, respectively.

Serum galactomannan antigen, a component of the *Aspergillus* cell wall, can be assayed for diagnosis of hematological involvement of disease.^[86]^ Use of this assay was reported in only 8 of 130 (6.15%) cases. Of those cases, the assay was positive in 5 of 8 (62.5%).^[14,16,59]^ In 2 of those cases, increasing levels of serum galactomannan antigen correlated with clinical signs of disease dissemination.^[16,59]^ No dissemination was observed in the other 3 positive cases.^[14]^

### 3.5 Multivariate model of disease mortality in immunocompromised patients

Factors that affected occurrence of mortality (in a positive or negative fashion) in univariate analysis with *P* < 0.2 were included in a multivariate linear regression model^[6]^ (Table 5). Patients with no diagnosed risk factors (otherwise known to be immunocompetent) were excluded from the model, resulting in a total n = 115. HIV/AIDS and disease dissemination were statistically significantly associated with greater mortality in this model of immunocompromised patients with PCA. The odds ratio (and 95% confidence interval) was 5.0736 (1.3781–29.4174) for HIV/AIDS and 5.3821 (1.7503–17.3991) for dissemination.

**Table 5:**
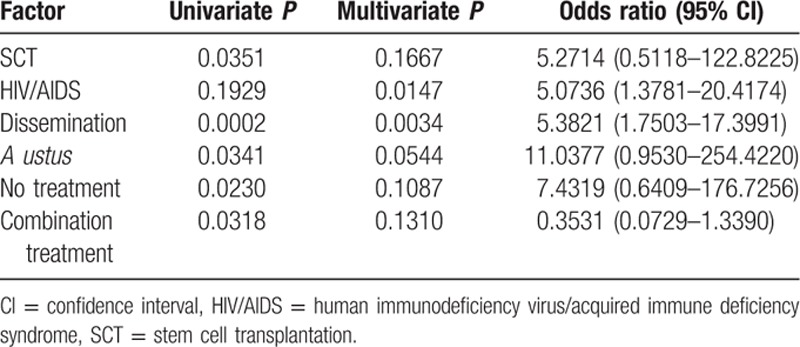
Multivariate model of factors on immunocompromised patient mortality.

## 4 Discussion

This is the largest and more comprehensive analysis of published PCA cases to date, composed of 130 patients. The largest previous database of PCA patients collected from the literature consisted of 14 patients and focused on underlying patient diseases, associated *Aspergillus* species, and histological features but not on outcome.^[14]^ In the current study, the age and sex distribution is consistent with other large collections of case reports in the literature of aspergillosis in general.^[87,88]^ However, the proportion of PCA patients with no apparent risk factors (15 of 130, 11.5%) in our study was higher than what has been reported in other forms of aspergillosis (e.g., pulmonary, sinus, cerebral, or bone/joint aspergillosis). It is possible that colonization of the skin is more common than other organ systems given its high exposure to environmental conidia; this is supported by our finding that cases of PCA in immunocompetent patients were associated with occupation (agricultural work) and, in some cases, local trauma preceding infection. Prior minor injury may have been underreported due to recall biases.

Aspergillosis remains a disease with high lethality. In other large collections of patient cases from the literature, mortality with disease for cerebral aspergillosis, sinus aspergillosis, pulmonary aspergillosis, and bone/joint aspergillosis has been reported as 88.1%,^[87]^ 65.6%,^[89]^ 60.2%,^[87]^ and 25%,^[88]^ respectively. In this study, mortality in patients with PCA was found to be 31.5% (or 35.7% with the exclusion of immunocompetent patients). Of note, none of the 15 cases of patients with presumed immunocompetence reported dissemination or death. In univariate analysis, systemic underlying conditions (often with accompanying immunodeficiencies) were significantly associated with increased likelihood of mortality (*P* = 0.0104). These findings further support the recommendation that reversal of immunosuppression is key to improving outcomes in *Aspergillus*-related disease.^[5]^

Only 42.3% of identified isolates were due to *A fumigatus* in our review of PCA (35.1% of identified isolates were *A flavus*). This is in contrast to other forms of invasive aspergillosis. For example, in 1 large collection of 261 cases of invasive aspergillosis (including pulmonary, skin, sinus, central nervous system, and disseminated cases), 66% of those identified were *A fumigatus* (14% were *A flavus*).^[90]^ It has been hypothesized that *A fumigatus* is the predominant pathogen associated with pulmonary aspergillosis (the most common form of aspergillosis^[87]^) due to the ability of its relatively smaller conidia to evade pulmonary clearance.^[91]^ As PCA occurs due to direct fungal inoculation after disruption of the epithelial barrier,^[2]^ conidia size differences between species of *Aspergillus* may have less relevance in developing primary skin infection. Understanding the increased distribution of species associated with PCA might be important for treatment, as resistance patterns differ between species; for example, *A terreus* is generally considered resistant to amphotericin B and *A ustus* has demonstrated increased azole resistance.^[5,92]^ In this study, *A ustus* was significantly associated with greater mortality in univariate analysis (*P* = 0.0341). However, as *A ustus* was involved in only 5 of 130 cases, further investigation is required.

The most current treatment guidelines discuss both surgical intervention and systemic antifungal therapy for cutaneous aspergillosis.^[5]^ In this study, univariate analysis indicated that a combination of systemic *Aspergillus*-active antifungal therapy and surgery was associated with significantly less mortality (*P* = 0.0230) and no treatment at all was associated with significantly greater mortality (*P* = 0.0318). PCA often results in a necrotic lesion, and *Aspergillus* has been demonstrated to both destroy blood vessels and mitigate host angiogenesis and wound healing.^[93]^ Combined, these factors may inhibit systemic delivery of antifungals to the wound bed. Therefore, reducing as much fungal burden as possible by surgical resection and debridement of the cutaneous lesion may synergistically improve patient outcome. Surgery alone was associated with significantly increased rates of recurrence (*P* = 0.0022). Others have observed that surgical treatment alone in the absence of immunocompetency is often ineffective in the treatment of cutaneous aspergillosis.^[94]^ Analogous to tumor resection, it is possible that surgery without systemic antifungal use may allow the survival of trace amounts of pathogen located at the wound margins or already spread distal to the wound through the vasculature or lymphatics. Surgical resection of large lesions was often associated with significant scarring and disfiguration.^[17,21,23,29,38,68,72,75]^ Ultimately, the optimal timing and extent of surgery remains unclear as patient selection is a confounding variable for better outcomes with surgery.

Angioinvasion and dissemination of *Aspergillus* to other organ systems are predictive of poor response to therapy and is associated with increased mortality.^[87,90]^ In this study, 59.1% of PCA patients with dissemination died and in univariate analysis, dissemination was significantly associated with mortality (*P* = 0.0002). Immune status and dissemination are clearly linked; there were cases in which immunocompetent patients in resource-poor settings had PCA lesions without treatment for over >10 years without disease dissemination.^[45,53]^ The most common site of dissemination was the lungs (70.0% of cases), but a wide range of organs have been reported. The time to dissemination varied greatly, with a median of approximately 1 month, standard deviation of 39.8 days, and range from 3 to 120 days. From a clinical standpoint, these data in sum (high lethality associated with dissemination and high variance in the timeline of dissemination development) support aggressive vigilance for dissemination throughout the treatment of PCA, especially as it may not be clear when a patient's primary lesion began developing. Serum galactomannan antigen was assayed in only 8 patients; in the 5 patients with a positive assay, only 2 had signs of disseminated disease. Based on these limited data, it is unclear if serum galactomannan antigen is useful for monitoring dissemination in PCA.

In our multivariate linear regression model of immunocompromised patients, HIV/AIDS (*P* = 0.0147) and disease dissemination (*P* = 0.0034) were significantly associated with increased mortality. In a previously published multivariate model of factors affecting mortality in immunocompromised patients with invasive aspergillosis in general, dissemination and underlying host disease were also significantly correlated with mortality (in addition to other factors such as creatinine clearance, steroid usage, and monocyte count).^[95]^ Of note, in multivariate analysis, no therapeutic modality demonstrated improved patient outcome compared to the others. Due to the complexity of *Aspergillus* infection pathophysiology (e.g., the aforementioned host angiogenic suppression),^[93]^ development of novel therapies that improve wound healing and reverse these disease phenotypes may be warranted.

### 4.1 Strengths and limitations

One of the main strengths of the study is the number of cases included that allowed us to develop a robust multivariate model that showed that dissemination of disease and underlying host diseases (HIV/AIDS) are significant in contributing to patient mortality. In addition, we were able for the first time in PCA to characterize key disease outcomes in addition to mortality, such as recurrence and dissemination. However, this study is not without limitations, chiefly because of publication, ascertainment, and classification biases. Due to the complexity of the patient population that is afflicted by PCA, it was hard to dissect PCA-attributable mortality. As occurs whenever patient data are collected from multiple studies across multiple decades, inconsistencies occur in the literature as medicine evolves. New diagnostic technologies (e.g., computed tomography, polymerase chain reaction, and galactomannan assays) as well as therapeutic modalities (e.g., the introduction of potent new triazoles such as voriconazole) were introduced between the first and last cases reported (1967–2015). Likewise, resistance patterns in *Aspergillus* species have also evolved over this time period and patient outcome associated with resistant species is poor.^[96]^ In 1 case series, several patients were diagnosed via histopathology but cultures were negative.^[24]^ This may introduce classification bias as other hyalohyphomycetes, such as *Fusarium*, have been mistaken as *Aspergillus* by histopathology.^[97]^ Cutaneous mucormycosis, another skin-based fungal disease accompanied by local necrosis, shares a similar clinical presentation to PCA but can be differentiated via immunohistological and/or molecular analysis.^[98,99]^

PCA diagnosis was made by evidence of a cutaneous lesion infected with *Aspergillus* in the absence of infection elsewhere. It is possible that some cases of PCA were in fact secondary cutaneous aspergillosis that had become disseminated from undetected lesions in the lungs or elsewhere. This possibility was largely excluded by lack of radiologic findings; however, it is impossible to definitely rule out spread from a nidus in the lungs too small to detect. Likewise, the time between PCA diagnosis and dissemination diagnosis may not be a reliable indicator of the timescale of dissemination, as it is not always clear when the primary lesion first developed or when dissemination actually occurred. Lastly, reporting on patient immune status as reflected by markers such as white blood cell count at different stages of disease would have complemented many of the findings in this study. However, given the variation of detail surrounding immunocompetence presented in the literature, these markers were not included in this analysis.

## 5 Conclusions

Although relatively uncommon, PCA is a condition associated with extensive morbidity and mortality and significant rates of disease recurrence and dissemination in a heterogeneous group of patients with varying degrees of immune suppression. Dissemination and HIV/AIDS status were significantly associated with increased mortality. Early diagnosis and prevention of dissemination of PCA is key to improved outcomes. Although further investigation is required, these findings suggest that aggressive therapy, stimulating immunocompetency, holding a high suspicion for dissemination even early in the disease process, and the development of new therapeutic modalities are of importance in the treatment of PCA.

## Acknowledgments

AMT would like to thank the Baylor College of Medicine Medical Scientist Training Program (NIH T32 GM007330) and the Barrow Scholars Program. The authors would also like to thank Ms Ying Jiang, MS, for her expertise in biostatistics.

## Supplementary Material

Supplemental Digital Content
