# Supplementary material for: Factors affecting patient outcome in primary cutaneous aspergillosis
Source: Medicine (Baltimore). 2016 Jul 1;95(26):e3747. doi: 10.1097/MD.0000000000003747 (PMC4937894; doi:10.1097/MD.0000000000003747)
Supplement: Supplemental Digital Content [file medi-95-e3747-s001.docx]

**Supplementary Table 1.** Characteristics of the examined literature

| **Literature Characteristics** | | |
| --- | --- | --- |
| Reported Cases |  | n |
|  | Total  Mean Cases Per Manuscript  Median Cases Per Manuscript  Standard Deviation  Range | 130 1.7 1 1.9  1-11 |
| Reported Cases Per Decade | Decade | N |
|  | 1960-1969 1970-1979 1980-1989 1990-1999 2000-2009 2010-2015 | 1  1 24 32 48 24 |
| Reporting Period |  | Year |
|  | Range | 1967-2015 |
